# Progression of Cystic Fibrosis Lung Disease from Childhood to Adulthood: Neutrophils, Neutrophil Extracellular Trap (NET) Formation, and NET Degradation

**DOI:** 10.3390/genes10030183

**Published:** 2019-02-26

**Authors:** Meraj A. Khan, Zubair Sabz Ali, Neil Sweezey, Hartmut Grasemann, Nades Palaniyar

**Affiliations:** 1 Translational Medicine, Peter Gilgan Center for Research and Learning, The Hospital for Sick Children, Toronto, ON M5G 0A4, Canada; meraj.khan@sickkids.ca (M.A.K.); zubair.sabzali@sickkids.ca (Z.S.A.); neil.sweezey@sickkids.ca (N.S.); hartmut.grasemann@sickkids.ca (H.G.); 2 Institute of Medical Sciences, Faculty of Medicine, University of Toronto, Toronto, ON M5G 1X8, Canada; 3 Division of Respiratory Medicine, Department of Paediatrics, The Hospital for Sick Children, and University of Toronto, Toronto, ON M5G 1X8, Canada; 4 Department of Physiology, Faculty of Medicine, University of Toronto, Toronto, ON M5G 1X8, Canada; 5 Laboratory Medicine and Pathobiology, Faculty of Medicine, University of Toronto, Toronto, ON M5G 1X8, Canada

**Keywords:** cystic fibrosis, cystic fibrosis transmembrane conductance regulator, lung disease, neutrophils, neutrophil extracellular traps (NETs), NETosis, DNase

## Abstract

Genetic defects in cystic fibrosis (CF) transmembrane conductance regulator (CFTR) gene cause CF. Infants with CFTR mutations show a peribronchial neutrophil infiltration prior to the establishment of infection in their lung. The inflammatory response progressively increases in children that include both upper and lower airways. Infectious and inflammatory response leads to an increase in mucus viscosity and mucus plugging of small and medium-size bronchioles. Eventually, neutrophils chronically infiltrate the airways with biofilm or chronic bacterial infection. Perpetual infection and airway inflammation destroy the lungs, which leads to increased morbidity and eventual mortality in most of the patients with CF. Studies have now established that neutrophil cytotoxins, extracellular DNA, and neutrophil extracellular traps (NETs) are associated with increased mucus clogging and lung injury in CF. In addition to opportunistic pathogens, various aspects of the CF airway milieux (e.g., airway pH, salt concentration, and neutrophil phenotypes) influence the NETotic capacity of neutrophils. CF airway milieu may promote the survival of neutrophils and eventual pro-inflammatory aberrant NETosis, rather than the anti-inflammatory apoptotic death in these cells. Degrading NETs helps to manage CF airway disease; since DNAse treatment release cytotoxins from the NETs, further improvements are needed to degrade NETs with maximal positive effects. Neutrophil-T cell interactions may be important in regulating viral infection-mediated pulmonary exacerbations in patients with bacterial infections. Therefore, clarifying the role of neutrophils and NETs in CF lung disease and identifying therapies that preserve the positive effects of neutrophils, while reducing the detrimental effects of NETs and cytotoxic components, are essential in achieving innovative therapeutic advances.

## 1. Introduction

Lung disease is the principal cause of morbidity and mortality in patients with cystic fibrosis (CF) [1,2]. The fundamental cause of CF is the recessive mutations in the Cystic Fibrosis Transmembrane Conductance Regulator (CFTR) gene, which encodes a chloride/bicarbonate (Cl^−^ and HCO_3_^−^) anion channel [3,4]. Defective CFTR of respiratory epithelium alters transepithelial ion transport, pH regulation, airway surface hydration, and mucociliary clearance [5,6,7,8,9,10]. Various infections (bacterial, fungal, viral), pro-inflammatory milieu, massive neutrophil influx, and subsequent neutrophil extracellular traps (NETs) release resulting in the accumulation of extracellular DNA and cytotoxic peptides cause persistent CF lung injury [11,12,13,14,15,16,17,18].

Neutrophilic infiltration of the airways occurs secondary to the presence of cytokines and chemokines, including interleukin−8 (IL-8), tumor necrosis factor−α (TNF-α), and IL−1β, complement-derived chemoattractants (e.g., C5a) and lipid mediators (e.g., leukotriene B4 and hepoxillins) [14,17,19,20,21,22]. IL-8 and lipid intermediates sustain the neutrophilic influx; TNF-α and IL-1β increase neutrophilic responses to chemoattractants; and, TNF-α stimulates neutrophilic pro-inflammatory secretory and oxidative burst activities [22,23,24,25]. Infiltrated neutrophils either phagocytose microbial pathogens and generate reactive oxygen species (ROS) for intracellular killing, or degranulate to release cytotoxic peptides for extracellular killing, or cast NETs as a DNA-mesh to ensnare microbial pathogens [26,27,28]. During NETosis, neutrophils release, decondensed chromatin coated with elastase, myeloperoxidase (MPO), and other cytotoxic granular proteases [12,27,29,30,31,32]. These neutrophil components perpetuate severe inflammation that characterizes CF lung disease [10,11,31,33,34,35]. Excess NETs increase the viscosity of endobronchial secretions, further hampering mucociliary clearance and eventual airway damage [11,12,35,36,37].

**Figure 1:**
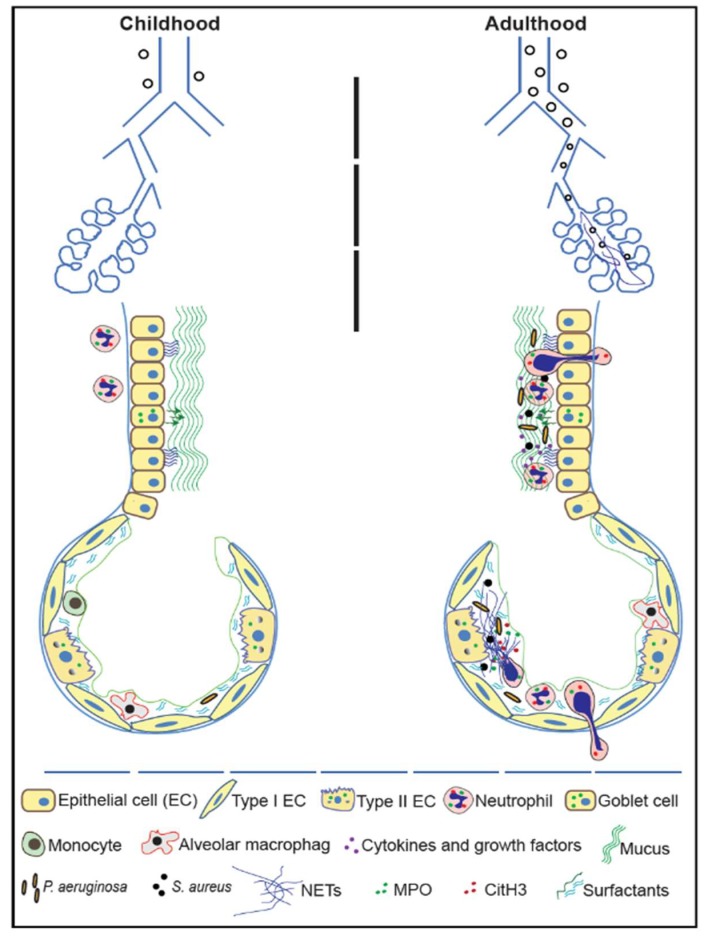
Airway alterations and neutrophil extracellular traps (NET) formation in Cystic Fibrosis airways. In fetal stages and early childhood, neutrophilic inflammation at the peribronchial regions is identifiable in patients with CF. In early childhood, CF airways have excess mucus and obstructive secretions, but not persistent bacterial infections. CF-associated airway abnormalities continue to increase throughout the childhood and include lower airway inflammation. During the disease progression, various microbial components and inflammatory cytokines and lipid mediators induce NETosis. Excess NETs and their cytotoxic components combined with thick and dry mucus exacerbate CF airway disease. Even with therapeutic interventions, the airway destruction/complications progress with age, resulting in irreversible lung damage, which often requires lung transplantation for survival. Minimizing excess NET formation, while preserving antimicrobial functions of neutrophil is a therapeutic option to minimize NET-mediated destruction of CF lungs.

Infants with CF can show a peribronchial neutrophilic infiltration prior to the establishment of an initial infection [10,12,17,32,38,39,40]. The inflammatory response increases gradually in toddlers and older children, with increasing mucus viscosity and mucus plugging of small and medium size bronchioles [8,41,42]. In older patients, increased levels of airway cytokines and chemoattractants (e.g., IL−8) further increase neutrophil influx, thus aggravating lung complications [13,43,44,45,46,47]. These inflammatory lung conditions facilitate chronic infection of select groups of bacteria at different stages of lung disease (Figure 1).

Children with CF that have *Pseudomonas aeruginosa*-mediated inflammation in the upper and lower airways have elevated neutrophil counts, levels of IL-8, and free neutrophil proteases. By contrast, *Staphylococcus aureus* infection is primarily related to lower airway inflammation [48,49,50]. Infections with both *P. aeruginosa* and *S. aureus* have additive effects, exacerbating inflammation in both the upper and lower airways [51,52,53,54]. Neutrophil-dominated inflammatory responses are observed more in the lower than the upper CF airways. *P. aeruginosa* and other pathogens that infect the lower airways enhance the persistence of the chronic inflammatory response, leading to persistent airway damage, requiring lung transplantation [48,49,53,55].

However, the survival of lung transplant recipients is low when compared to other solid organ transplantations, and it is often complicated by acute cellular rejection, infections, and the development of chronic lung allograft dysfunction (CLAD) or bronchiolitis obliterans (BO), and occasionally post-transplant lymphoproliferative disease (PTLD). Immunosuppressive therapy is used for minimizing rejection risk and antibiotics are used for the treatment of bacterial infections [56].

Airway infection, cytokines, and airway surface liquid (ASL) pH affect the bactericidal activity of neutrophils and NETosis [57,58,59,60,61]. Acidification of the CF bronchoalveolar environment has been reported [60]. Therefore, a detailed understanding of the molecular mechanisms of NETosis (including the effects of reduced pH, metabolic pathways, kinases, sodium/potassium channels, reactive oxygen species (ROS), and transcription regulation) would help to identify potential therapeutic targets to control the excess NETosis without altering the anti-microbial functions of neutrophils in CF [21]. Screening FDA-approved drugs on CF neutrophils will be helpful in delineating NETosis mechanisms and identifying the potential NETosis regulating drugs for treating CF lung disease.

## 2. Cystic Fibrosis

CFTR is expressed in multiple organs, including the airways (e.g., epithelial cells), pancreas (e.g., β cells, duct epithelial cells), and innate immune cells (e.g., neutrophils) [62,63]. Different CFTR mutations with varying effects on these cells have been identified [64,65,66]. Recent studies show that a rare cell population (pulmonary ionocytes; ~1% of epithelial cells) is responsible for expressing most (>90%) of the CFTR protein in the airways. Ionocytes in other organisms are responsible for regulating pH [67]; hence, these cells may be very important for regulating pH and chloride transport in human airways. The presence of CFTR in many organs makes it a systemic regulator of various functions. Therefore, the dysfunctional CFTR disrupts many physiological processes, resulting in diverse health complications [62].

### 2.1. Cystic Fibrosis Transmembrane Conductance Regulator

Cystic Fibrosis Transmembrane Conductance Regulator is an ATP-binding cassette (ABC) protein [68], and it has two transmembrane domains, each with a cytoplasmic nucleotide binding domain (NBD1 and NBD2). Unlike typical ABCs, the CFTR has a regulatory domain (R-domain) that connects the 2-transmembrane domains [69]. Interestingly, the R-domain is regulated through phosphorylation by cAMP-dependent protein kinase A (PKA). The R-domain phosphorylation and interaction with NBD are vital for channel activity [70]. The dephosphorylation of the R domain inhibits channel opening. However, CFTR molecules lacking the R domain and distal NBD1 that has a deletion of three amino acid residues (634–836) show comparative channel opening rates in the absence of PKA [62,70]. This suggests that R-domain phosphorylation of CFTR allows for ion conduction, whereas the lack of phosphorylation results in channel closing.

### 2.2. Classes of Cystic Fibrosis Transmembrane Conductance Regulator Mutations

The *CFTR* mutation severity can influence the age of diagnosis, onset of digestive symptoms, and peripheral blood oxygenation. Over 2000 different mutations have been identified [65,66,71,72] and subsequently organized under six different classes (Table 1). Class I are nonsense mutations with large deletions that lead to premature stop codons (e.g., R553X), resulting in a truncated CFTR protein. Class II mutations lead to improper folding and the transport of the protein to the cell surface because of missense mutations and in-frame deletions (e.g., F508del). Approximately 90% of patients with CF in Canada have a copy of the F508del (class II) mutation. In addition, 48.6% of the total CF population have two copies of the F508del mutation [72]. Therefore, identifying effective therapeutic options for treating F508del associated dysregulations in CF is of high importance. Class III mutations lead to a gating defect in CFTR membrane protein due to missense mutations, also known as a gating mutation (e.g., G551D) [69,73,74]. Recent clinical studies show that CFTR potentiators (e.g., Ivacaftor) successfully correct these defects in patients with these mutations [75,76,77,78]. Class IV mutations lead to disruptions in the chloride conducting capacity of the CFTR protein because of missense mutations and protein misfolding (e.g., R117H) [79,80]. Class V mutations include missense mutations that lead to a decrease in CFTR protein synthesis, resulting in a low abundance on the cell surface (e.g., A455E) [81]. The cell surface CFTR proteins are quickly removed and degraded (high turnover) in Class VI mutations, leading to the lack of normal CFTR function (i.e., 120del123) [71,82]. The inadequate hydration of the airway mucus layer and impairment of the mucociliary movement is in consequence to severe CFTR mutations (i.e., F508del), which can result in subsequent CF that is related physiological complications [71,83].

### 2.3. Cystic Fibrosis Disease Pathophysiology: Mucus Dehydration and Declining Lung Function

Mucous dehydration plays an important role in CF acute lung disease exacerbation and pulmonary complications. Airway epithelial cells express CFTR and epithelial sodium channel (ENaC) [88]. CFTR typically inhibits ENaC opening. However, the deficiency or reduced expression of CFTR fails to inhibit the ENaC opening [89,90]. The accumulated Cl^−^ and Na^+^ ions inside the cell form an osmotic gradient, resulting in the dehydration of the ASL [88,91]. The dehydrated ASL, impairs ciliary movement and the subsequent mucus clearance from the CF airways [88,92]. Human CF bronchial cell cultures and mouse models (overexpressing the β subunit of ENaC) show increased sodium absorption and ASL dehydration [90,93,94]. Adenosine cannot regulate ASL if CFTR is defective, and ATP alone regulates ASL through ENaC and calcium-activated chloride channels (CaCC) [95,96,97]. Normal hydration of the mucus layer in the airways allows for effective mucociliary clearance [88]. However, human CF airways inevitably face ASL dehydration, which is believed to be the primary cause of mucociliary clearance failure, ultimately predisposing patients to the increased risk of chronic infection and mortality [73,83,88].

Increased mucus viscosity in CF airways is believed to derive from several factors. Reduced HCO_3_− secretion enhances calcium-mediated mucin crosslinking and consequently mucus thickening [98,99]. Mucus thickening can also be a direct effect of ASL dehydration, which concentrates mucins and amplifies mucin crosslinking [92,100]. Additionally, CF airways accumulate large amounts of DNA (derived from neutrophils) and actin, which further increase mucus viscosity [1,101]. Although T cells are not present in the airway lumen, many CF patients have a helper T-cell 2 (Th2) predominant cytokine profile, with an abundance of IL-4, IL-9, and IL-13 cytokines in the sputum [25,102,103,104]. Th2 cytokines IL-4 and IL-13 may contribute to the disease pathophysiology by inducing goblet cell metaplasia/hyperplasia and mucin production [13,22,105,106]. Th17 cytokine predominance in CF airways is considered to increase neutrophil recruitment [105,107,108,109]. Conversely, a recent study suggests that neutrophils release arginase, which deplete arginine and prevent T-cell expansion [110]. This may be a reason why T cells are not detected in the neutrophil-rich CF airway lumen. Although mucus dehydration is a well-known contributor of lung function decline in CF patients, the accumulation of neutrophils and NETs can increase mucus viscosity and promote pulmonary exacerbations.

### 2.4. Infection and Inflammation in Cystic Fibrosis

Cystic fibrosis airways are characterized by inflammation and recurrent/chronic bacterial infections, leading to the fatal decline of pulmonary function and eventually respiratory failure [23,38]. Notably, early signs of lung inflammation can precede colonization and infection [23,48,111,112,113]. Pro-inflammatory markers, such as human neutrophil elastase, IL-1β, IL-8, and TNF-α, are elevated in the CF airway milieu [31,114]. Perhaps the most convincing evidence supporting inflammation as a primary event comes from studies that were conducted on CF fetuses. These studies show that increased proinflammatory cytokines can already be found in CF airways during fetal development [23,114]. Although newborn infants with CF are generally thought to have normal lungs, an autopsy of CF fetuses report prenatal abnormalities in the airways, including the loss of microvilli on airway epithelial cells and the dilation of tracheal submucosal gland duct [115]. Furthermore, the inflammatory response to bacteria (expressed as the ratio of neutrophils or IL-8 to bacteria) is exaggerated and disproportionate in children with CF when compared to non-CF control patients [1,114,116].

Nevertheless, there are studies that argue inflammation as a secondary event to infection [117,118]. The reasons for the inconsistency between the studies may be related to differences in methodology and experimental conditions. However, there is a general agreement regarding the events occurring during disease progression. CF small airways are the initial and major sites of lung disease [88]. Early lung structural abnormalities (e.g., bronchiectasis, air trapping) can be detected in infants with CF by CT scan within the first few months, even in the absence of any clinically apparent lung disease [119]. Progressive structural lung disease in infants with CF is associated with neutrophilic inflammation and pulmonary infections [14,39,120]. Pulmonary inflammation reduces lung function and pulmonary infection exacerbates the decline in lung function [24,118,121,122,123].

## 3. Neutrophils

Neutrophils play a pivotal innate immune defense role against infectious and facultative microbial pathogens and they are often referred to as the first line of defense against infection. They constitute ~60% of all leukocytes in human blood [124,125]. There are more neutrophils in the pulmonary capillaries than in the systemic circulation, facilitating a rapid entry into the lungs in response to infection and inflammatory stimuli [15,125,126].

Neutrophils are activated upon interaction with microbial pathogens, inflammatory cytokines, and sterile inflammatory agents [30,127,128,129,130]. When activated, neutrophils undergo distinct intracellular signaling, which can dictate their responsive behavior. Neutrophils kill microorganisms directly by phagocytosis or degranulation, and indirectly by releasing pro-inflammatory cytokines (e.g., TNF-α, IL-1β, CC, and CXC), chemokines (e.g., IL-8, IP-10, MIP-1α), and immunoregulatory cytokines (e.g., IFN-γ, IL-4, IL-10, IL-13) [131,132,133]. These secreted factors stimulate the production of downstream factors and recruit more neutrophils or other leukocytes to the site of infection or inflammation [39,134]. Importantly, these cytokines also regulate neutrophil function and NETosis [135].

### 3.1. Neutrophil Development and Maturation

Neutrophil development occurs in the bone marrow, followed by full maturation in the peripheral blood over time [136]. Every day, approximately 50–100 billion neutrophils are produced in the bone marrow and one-billion released into circulation [126,137]. Neutrophils, however, are short-lived cells with a half-life of approximately 8 h in circulation. The neutrophil release into the bloodstream from the bone marrow can be attributed to a variety of cytokines, such as the granulocyte colony stimulating factor (G-CSF) and CXC-motif ligand chemokines (e.g., CXCL1) [124]. The neutrophil chemokine receptor for CXCL4 (CXCR4) and its binding to its ligand stromal derived factor 1 (SDF-1/CXCL12) on the bone marrow stromal cells restricts the neutrophil release into circulation [136]. The chemokine G-CSF down regulates the neutrophil cell surface presence of CXCR4, promoting neutrophil flow into circulation [124]. Developing neutrophils express elevated levels of CXCR4 [138]. Mature neutrophil expresses less CXCR4 and more CXCR2 (IL-8 receptor) [139,140]. Neutrophil can undergo senescence, a period of unresponsive behavior to chemoattractants, suppressed ROS production, and degranulation. Senescent neutrophils have an abundance of CXCR4, which is suggestive of neutrophils transportation back to the bone marrow that is to be cleared by resident macrophages [126,141]. Interestingly, mature neutrophils from CF patients show varying CXCR4 profiles. Peripheral blood mature CF neutrophils express reduced levels of CXCR4. However, they express significantly elevated cell surface levels of CXCR4 as they extravasate into the airway secretions [142]. Furthermore, granulocytes in CF airways with chronic fungal colonization by *Aspergillus fumigatus* also express high levels of CXCR4 when compared to healthy controls [143]. The CXCR4 expression profile in CF neutrophils may highlight the importance of the receptor as an avenue for further investigation.

### 3.2. Cystic Fibrosis Transmembrane Conductance Regulator in Neutrophils

Neutrophils express CFTR on their cell surface and on phagolysosomal membranes [144]. Interestingly, the differentiations of human leukocyte 60 (HL60) promyelocytic cells into neutrophil like cells result in increased CFTR expression [21,145]. This may suggest that CFTR has a role in mature neutrophils. Given its function as a chloride ion transporter, studies suggest a vital role for CFTR in supplying phagolysosomes with chloride ions for use in bacterial killing. The phagolysosome is an organelle that encapsulates foreign pathogens and it is fully formed shortly after phagocytosis [144]. NADPH Oxidase II (NOX-2) in the phagolysosomes generates reactive oxygen species, including hydrogen peroxide (H_2_O_2_). Myeloperoxidase (MPO) entering the phagosomes catalyzes the conversion of H_2_O_2_ and Cl^−^ to create hypochlorous acid (HOCl; active ingredient of bleach), which is a crucial component in phagolysosome acidification, chlorination of microbial components, and bacterial degradation [29,32,144]. Bacterial chlorination in neutrophils can be detected 5 min post-phagocytosis and it progressively increases for the following 60 min [146]. The presence of the CFTR and the corresponding antimicrobial mechanisms in the neutrophil suggests a critical role in its fight against pathogens, which may be compromised in CF patients. The different classes of CFTR mutations may exert varying effects on neutrophil dysfunction. Baseline changes in neutrophils functions in CF patients have been reported. Ineffective neutrophil functions, including bacterial killing due to different CFTR genotypes, may add to lung complications in CF patients [75,147].

### 3.3. Cystic Fibrosis Neutrophil Dysfunction

The paradox in CF lung disease is that plenty of neutrophils are present in the airway lumen but they are unable to effectively control bacterial infection. Neutrophils in CF airways exhibit a dysfunctional phenotype that deviates from their wild type counterparts [148]. A study by Zhou et al. revealed defective HOCl production and bacterial chlorination in CF neutrophils [144]. CFTR knockdown in HL60 cells showed impaired neutrophil-mediated killing of bacteria, suggesting a vital role for CFTR in killing infectious pathogens [21,145]. When compared to healthy neutrophils, CF neutrophils display increased survival because of the suppression of apoptosis in these cells. Interestingly, a delay in apoptosis correlates with the loss of CFTR function, but not in inflammation. The difference in the CF neutrophil viability may be important in the inherent innate immune dysfunction [17,147]. Interestingly, peripheral blood CF neutrophils produce significantly more ROS than the healthy control neutrophils [149]. However, tissue location influences ROS production: airway neutrophils release less ROS than peripheral blood neutrophils [150]. Various dysfunctions of CF neutrophils can significantly compromise their ability to perform anti-microbial functions. This deviation can be lethal for CF patients in their fight against foreign pathogens.

## 4. Neutrophil Anti-Microbial Properties

Neutrophils have many anti-microbial mechanisms. Neutrophils can perform phagocytosis and the subsequent digestion of pathogens and cell debris [151,152,153]. Internalized pathogens are contained in vacuoles, called phagosomes, where antimicrobial peptides from granules and reactive oxygen species (ROS) that are produced by NADPH oxidase work together to create an environment that is toxic for most pathogens [154]. Neutrophils also undergo degranulation, releasing toxic ROS and antimicrobial granular proteins into the extracellular space [132,155,156,157]. The granules contain antimicrobial proteins (e.g., myeloperoxidase, lysozyme, lactoferrin, elastase, defensins, gelatinase, cathelicidins, and cathepsins) that help in fighting infection. In addition, neutrophils are able to undergo a unique form of cell death, called NETosis [27,132,158,159,160].

### 4.1. Neutrophil Extracellular Trap Formation (NETosis)

In response to certain infectious agents, neutrophils can expel their cellular DNA coated with antimicrobial granular proteins to the extracellular environment. The key events of NETosis include elevated intracellular calcium, ROS production (by NADPH oxidase; NOX and/or mitochondria), kinase activation (e.g., ERK, p38, JNK, Src, Akt, etc), DNA decondensation (via histone-3 citrullination, acetylation, and transcriptional firing), and eventual NET release [30,127,161,162,163,164,165]. The net-like structure is excellent for capturing microorganisms, including bacteria, fungi, and viruses [135,166,167,168]. Although NETs may serve as a host defense structure, they can have deleterious effects on the surrounding tissues. CF sputum and bronchioalveolar lavage have higher than normal concentrations of cytotoxic/anti-microbial peptides and granular components (such as MPO and elastase) [169]. Their levels positively correlate with the degree of lung disease severity, implicating NETosis or other lytic forms of neutrophil death in the pathogenesis of the disease [18,33]. Previously, necrosis was considered to be the main form of cell death that contributes to free neutrophil DNA in CF the lungs. However, this notion has been revised after the discovery of NETosis, and by the presence of MPO, elastase, granular peptides, and DNA as NETs in CF sputum and bronchioalveolar lavage samples [169,170]. Furthermore, the presence of large quantities of DNA (earlier thought of from dying neutrophils, but now evidences are there to support that these DNA is from NETotic neutrophils), aggravating the mucus viscosity in CF airways supports the role of NETs and extracellular DNA in disease pathophysiology [33,36]. A recent finding of NETs that are likely induced by *P. aeruginosa* in CF sputum provides supportive evidence for the NETosis-mediated release of inflammatory mediators in CF airways [18,171,172].

**Figure 2:**
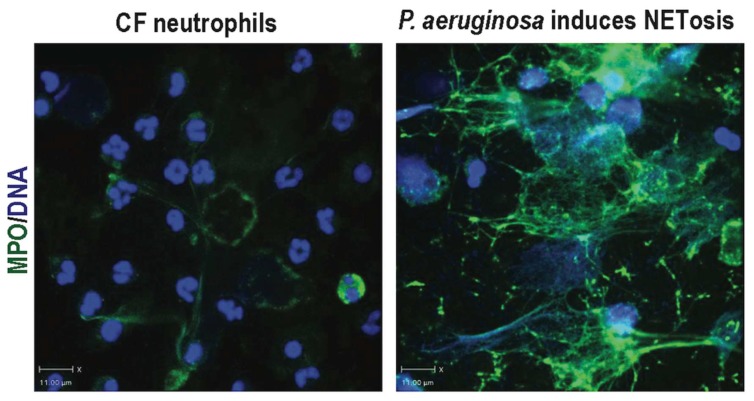
*Pseudomonas aeruginosa*, induces NETosis in CF neutrophils. Neutrophil purified from patients with CF has typical polymorphonuclear morphology (blue stain). Upon incubation of these neutrophils with *P. aeruginosa* (e.g., multiplicity of infection of 20), CF neutrophils readily release NETs-DNA (blue stain) coated with granular proteins (e.g., myeloperoxidase; immunostained with green fluorescence antibodies).

NETosis can be stimulated by infectious and inflammatory agents (e.g., higher numbers of bacteria per neutrophil, high concentration of lipopolysaccharide or LPS, pyocyanin, protozoa, fungi, viruses) or host-derived factors, such as granulocyte/macrophage colony-stimulating factor (GM-CSF), complement component 5a (C5a), activated platelets and singlet oxygen molecules as a result of infection, and inflammation [127,161,166,173,174,175,176]. Several-infection and inflammation-activating agonists generate diacyl glycerol (DAG) as the intercellular signaling intermediate. Hence, a diacyl glycerol mimetic, phorbol myristate acetate (PMA), is routinely used for inducing NETosis. PMA and several other microbial components activate protein kinase C and robustly induce NETosis [30,127,162,163,177]. Dysregulated NET formation or clearance has been associated with CF, chronic inflammatory, and autoimmune disorders [161]. The cause(s) and temporal onset of NETosis in CF airways are still not clearly established. As the lungs of CF patients are chronically colonized and/or infected with bacteria, it is reasonable to postulate that bacteria or their components may induce NETosis (Figure 2). Common pathogens (e.g., *Staphylococcus aureus, Pseudomonas aeruginosa,* and *Aspergillus fumigatus*) that colonize CF airways have been shown to be effective inducers of NETosis [173,178]. However, similarities/differences in the molecular mechanism of NETosis in healthy and CF neutrophils must be identified to devise specific therapeutic targets that are relevant to CF. To date, two distinct pathways (NOX-dependent and -independent) have been identified that mediate NET formation in infectious and non-infectious conditions in ex vivo and in vitro models [30,162,163,179].

#### 4.1.1. NOX-Dependent NETosis

Specific microbial pathogens (e.g., bacteria, such as *P. aeruginosa*, *Klebsiella pneumoniae,* and *S. aureus*; fungi, such as *Aspergillus fumigatus* and *Candida albicans*; viruses, such as cytomegalovirus and influenza), bacterial components (e.g., LPS), or certain pharmacological agents (e.g., PMA) induce neutrophil NADPH oxidase (Nox) [30,127]. Inside the phagosome, electrons that are generated by Nox react with oxygen to produce various ROS and other intermediates (e.g., H_2_O_2_, HOCl, NO, and singlet oxygen). Singlet oxygen is a member of the ROS family that has been shown to be essential in the formation of NETs [132,154,180]. Depending on the microbial load and the type of stimulus, neutrophils can switch from phagocytosis to NETosis. Phagocytosis and NETosis utilize several similar signaling events, but during NETosis, neutrophil granular contents enter nuclei, in addition to the phagosomes. Inside the nuclei, elastases and MPO bind chromatin, and they cleave and modify histones, respectively [161]. These events and transcription help to decondense the chromatin. Cytotoxic DNA-protein complexes are eventually released from the nuclei into the extracellular space as NETs. In addition to superoxide, Remijsen et al., (2011) showed that autophagy is also required for the generation of NETs [181]. Emerging evidence shows that the Nox-dependent NETosis pathway requires cell signaling involving the RAF-MEK-ERK kinase pathway [179]. A detailed understanding of the mechanisms that are involved in Nox-dependent NETosis should be useful in formulating a therapeutic strategy for regulating NETosis in CF airways in the future.

#### 4.1.2. NOX-Independent NETosis

Sterile injury can also induce NET [30,182]. Various pharmacological agents (e.g., calcium ionophores, such as A23187), microbial secretions (e.g., ionomycin), cytokines, inert particles (e.g., uric acid crystals), lipid intermediates (e.g., platelet activating factor or PAF, hepoxilins), contributions to the induction of Nox-independent NETosis can induce an increase in intracellular calcium [20,30,183,184,185,186]. Increased calcium levels activate calcium dependent potassium channels (e.g., SK3), generate mitochondrial ROS, and translocate the peptidylarginine deiminase 4 (PAD4) enzyme into the nuclei. PAD4 enzyme citrullinates histones (converting positively charged arginine into non-charged citrulline) to destabilize the interaction between negatively charged DNA and histones. This process helps to decondense chromatin and release cytotoxic NETs into the extracellular space. In general, the Nox-independent pathway is considered to be responsible for sterile injury-related NETosis [30,187]. Interestingly, CF neutrophils are observed to have dysfunctional mitochondrial ROS production and elevated intracellular calcium (mitochondrial leakage) [188,189]. These inherent characteristics of CF neutrophils resemble the initial steps of the NOX-independent pathway of NETosis. Therefore, CF neutrophils may be more susceptible to undergoing NETosis via the NOX-independent pathway. Certain agonists (e.g., LPS, bacteria, and hepoxilin lipid intermediates) can induce both Nox-dependent and independent NETosis [20,183]. Hence, CF neutrophils may be more prone to undergoing NETosis via both of the NETosis pathways [189]. Regardless of the type of NETosis (Nox-dependent or–independent), NETs can serve beneficial functions in trapping and killing infectious agents and of containing pro-inflammatory agents in the scaffold [30,181]. Nevertheless, excessive NETosis appears to contribute to a vicious cycle of dysregulated inflammation and infection that aggravates and perpetuates the lung disease in patients with CF [11].

#### 4.1.3. NETs and the Vicious Cycle of Cystic Fibrosis lung Disease

Cystic fibrosis airways are progressively damaged due to severe inflammation that is caused by proteases, cytotoxic compounds, oxygen free radicals, and possibly by excessive NET formation. Airway epithelial cells, host immune cells, and bacterial infection exacerbate the inflammation and the resulting damage. A deficient CFTR function in the epithelial cell causes abnormal signaling, with aberrant chemoattractants and cytokine production, dysregulated transcription factors and inflammatory mediators, and an environment that is conducive to microbial infection [1,45,48,190]. The DNA that is found in CF airways has been traditionally attributed primarily to necrotic neutrophils, but it has recently been suggested that the DNA is derived from NETs [33,36,170,171]. The presence of large quantities of cytotoxic peptides and granular proteins of NETs is deleterious to the surrounding tissues. NET accumulation in the CF airways as a result of dysregulated NETosis and their lack of clearance can severely compromise respiratory health. Vast amounts of free DNA accumulate in CF lungs, contributing to increased mucus viscosity [8,36,171,191].

NETs have been identified as contributing (NET-DNA aggravates mucus dehydration and airway clogging, while released cytotoxic peptides causes tissue injury) in different conditions of cystic fibrosis, acute lung injury, and infections [161]. Along with the neutrophil infiltration and inflammation, infection is also an important feature of CF. The microbiota that are present in CF airways are diverse, but chronic pulmonary infections are mainly dominated by opportunistic pathogens, including *P. aeruginosa* and *Burkholderia cepacian* [192,193]. In addition to the inability of neutrophils and NETs to eradicate the bacteria, DNA that are released from neutrophils also promote bacterial colonization and biofilm formation [194]. Neutrophils from CF and healthy human subjects are equally able to cast NETs that are induced by *P. aeruginosa* [195]. However, clinical strains of *P. aeruginosa* can acquire resistance to NET-mediated killing over the course of infection in CF airways [161,195]. Inflammation and massive neutrophil infiltration occurs early, even prior to any apparent infection in CF airways [151]. The CF airway milieu has an imbalanced cytokines profile and has been shown to increase NF-κβ activation and inflammatory cytokines, such as IL-8, TNF-α, and GM-CSF [129,131,196]. The effects of these host-derived molecules on NETosis in CF are not clearly known. All-in-all, the data suggest that there is a massive influx of neutrophils and release of neutrophil DNA (e.g., NET DNA) in the bronchioles of CF lungs that exacerbate the disease pathology by increasing the mucus viscosity and providing a better environment for the better colonization of infectious bacteria. Neutrophils of the CF patient (G551D) over the treatment of ivacaftor showed diminished activation, suggesting mutation specific functional modulation [75]. The improved neutrophil function with reduced NETotic propensity may help to reduce the inflammation and disease exacerbation. Collectively, these facts argue for a need to regulate neutrophil activation, augmenting of the clearance of NETs, and understanding that the mechanism of NETosis in association with CF could help to identify new effective therapeutic options.

## 5. Clearing Neutrophil Extracellular Traps (NETs)—DNases and Macrophages

Deoxyribonuclease (DNase) digests polymerized DNA; hence, it reduces mucus viscosity and promotes mucus clearance in the CF airways. Therefore, the use of aerosolized recombinant human DNase I (dornase alfa) is an attractive approach to alleviate DNA-related mucus viscosity in patients with CF [197]. One caveat; however, is the inhibition of the enzyme by globular actin. Since airways of CF patients contain an abundance of DNA and actin, this issue requires attentiveness. There are multiple investigations looking at DNases that are immune to actin-mediated inhibition. Chemically modified plant sourced DNase 1 “alidornase alfa” has been developed, which show the resistant to actin inhibition. A clinical study showed that alidornase alfa improved lung function when compared to baseline or treatment with recombinant human dornase alfa. There are three secretory enzymes, such as DNase1, DNase1-like 2, and DNase1-like 3, which cleave DNA [198]. Interestingly, DNase 1 and DNase 1, like 3, degrade the majority of the NETs [199]. Unlike its counterpart DNase1, DNase 1, like 3, is resistant to actin [200]. This makes the protein an attractive component for NET degradation in the airway. Although the commonly used dornase alfa has its caveats in clearing NETs, current investigations that aim to overcome this issue by modifying the DNase show promise in its effective use as a therapeutic approach [200,201].

Neutrophils in CF lungs also release extracellular proteases that destroy lung tissue, and exogenous protease inhibitors are ineffective in inhibiting these proteases. DNase can disrupt the ultrastructure of NETs, but DNase treatment can also dramatically increase the proteolytic activities of neutrophil enzymes (NE, cathepsin G, protease 3) that are bound to NETs. Ultimately, NETs or partially cleaved NET fragments can sequester active proteases [170]. The unresolved clearance of these NET-protein complexes may potentially give rise to an autoimmune response in CF patients.

Macrophages are also known to mediate NET clearance [202]. Monocytes are leukocytes that can differentiate into macrophages and dendritic cells in the lungs [203]. Unlike neutrophils, the chemoattractant activation of β1 and β2 integrin’s for chemotaxis is defective in monocytes cells that were isolated from CF patients [204]. This was also replicable in healthy monocytes that were exposed to the CFTR inhibitor CFTR (inh)-172. Interestingly, β1 and β2 function was restored when CF monocytes were treated with CFTR correctors VRT325 and VX809. In CF-like murine lung inflammation models, the monocytes were abundant in the lung parenchyma but not in the bronchoalveolar lavage [205]. This suggests that, in application to the CF condition, integrin-independent monocyte recruitment is normal, but transmigration into the alveolar space is impaired. The abundance of airway neutrophils and NETs, and the fact that macrophages can clear NETs, the lack of monocytes in the airway suggests the suppressed clearance of NETs, which is attributed to the excessive presence of extracellular DNA in CF sputum.

## 6. Suppressing NETs: Future Directions and Therapeutic Potential

The current understanding of the CF pathophysiology emphasizes a dire need to suppress inflammation and infection in CF lung disease. For that, the improved mucociliary movement, clearance of pathogens, and pro-inflammatory agents will be needed. Current developing therapies that are used for targeting NET-DNA in inflammatory lung diseases include DNase, anti-histone antibodies, and anti-proteases [170,181,200]. Dubois et al. showed that treating CF sputum with DNase can increase elastase activity [170]. Chronic inflammatory lung diseases already have elevated levels of proteases, which lead to lung damage and increased inflammation. Antiproteases are used in therapy to suppress the activity of these proteases [206]. The use of exogenous protease inhibitors alone has been shown to be ineffective in CF sputum, because NETs serve as a reservoir of these active proteases and protect them from inhibition [170]. The use of anti-histone antibodies has also shown to be protective of NETs-mediated lung damage in a Transfusion-related acute lung injury (TRALI) mouse model [207]. However, the use of anti-histone antibodies is highly immunogenic, inducing the production of autoantibodies and potentially promoting autoimmunity [11,169,208].

*Pseudomonas aeruginosa* biofilms protect the pathogen from immune system [209]. The NET-DNA with biofilm alginate in the CF airway also contributes to mucus viscosity and respiratory exacerbations [194]. Moving forward, the identification of novel therapeutic approaches that target the biofilm architecture and promote transformation to the vulnerable planktonic form will be important in the fight against CF lungs infections [210]. Overall, the currently available therapies can reduce some symptoms and slow lung disease progression, but they are insufficiently effective and have significant associated adverse effects. The development of new early and preventative therapies is needed to prevent tissue destruction.

Detailed information in CF models about the molecular mechanism of NETosis, operating pathways, involvement of kinases, sodium potassium channels, ROS, transcription factors, and the effects of modulating pH will inform efforts to identify potential targets to control the excess NETosis without impairing the required beneficial immune functions of the neutrophil. Identifying the drugs suppressing NETosis (both Nox-dependent and –independent pathways) without hampering antimicrobial activity will be helpful in delineating the mechanism of drug action and the role of NETosis in CF inflammation.

Alteration of the normal pH has been reported in the bronchoalveolar environment of the CF lung. The neutrophil intracellular pH is lower than the normal tissue pH, suggesting that excessive neutrophil infiltration may also lower overall CF bronchiolar pH. Intracellular pH also plays an important role in the activation of different neutrophil granular enzymes, including MPO and elastase. It will be important to study the effects of modulating pH during NETosis in the context of CF.

Cystic fibrosis airways are L-arginine deficient [211]. The CF airway neutrophil count correlates with arginase levels, and increased arginase levels are considered to be responsible for T-cell suppression [110]. The dysregulation of the T cell response in the CF airways may play an important role in the virus-related exacerbation of bacterial infections. Understanding mechanistic details and potential therapeutic approaches to regulate T-cell-neutrophil interactions could also help in reducing pulmonary exacerbations in CF airways.

## 7. Conclusions

In summary, neutrophils are the first line of defense against harmful agents. In CF, neutrophils are identified as vital innate immune cells, infiltrating and controlling chronic infection in the airways. The expression of defective CFTR in CF neutrophils is partly responsible for the disabled microbial clearance and perpetual airway inflammation and destruction. The massive neutrophil recruitment, infiltration, and NETosis in the CF airways have been attributed to worsening lung conditions. The DNA and cytotoxic granular components contribute to mucosal clogging and sterile damage, respectively. Although DNase and protease inhibitors are currently used in neutralizing this issue, the complete etiology of NETs in the worsening CF airway is still not clear. Therefore, understanding various molecular mechanisms and regulating key aspects of NETosis in the CF airway are crucial in identifying effective novel therapeutic approaches for treating CF lung disease.

## Figures and Tables

**Table 1:** Classes of *CFTR* mutations and molecular mechanisms.

Class	Molecular Mechanism	Mutation Examples
1	Non-sense mutation: premature stop codon → defective protein synthesis (no CFTR expression)	R553X, G542X [84]
2	Missense mutation: either (1) Misfolded CFTR protein and, or (2) not transported to the destination (or if so, only in residual amounts)	F508del (Most prevalent), N1303K [69,72]
3	Missense mutation (AA substitution): reduced of lack of CFTR opening in response to agonists → gating defect	G551D (Ivacaftor corrects), G1244E [69,74]
4	Missense mutation (AA substitution): restrict Cl^−^ transport across the channel → conductance defect	R117H, R334W [85,86]
5	Splicing defect: improper processing of CFTR mRNA → less CFTR protein abundance on the cell surface but the proteins are normal	A455E (Least prevalent) [81]
6	CFTR is functional, but unstable due to rapid removal and degradation	N287Y [87]

mRNA: messenger RNA.
